# Exogenous ketosis impacts neither performance nor muscle glycogen breakdown in prolonged endurance exercise

**DOI:** 10.1152/japplphysiol.00092.2020

**Published:** 2020-05-14

**Authors:** Chiel Poffé, Monique Ramaekers, Stijn Bogaerts, Peter Hespel

**Affiliations:** ^1^Exercise Physiology Research Group, Department of Movement Sciences, KU Leuven, Leuven, Belgium; ^2^Department of Physical and Rehabilitation Medicine, University Hospitals Leuven, Leuven, Belgium; ^3^Locomotor and Neurological Disorders, Department of Development and Regeneration, Faculty of Medicine, KU Leuven, Leuven, Belgium; ^4^Bakala Academy-Athletic Performance Center, KU Leuven, Leuven, Belgium

**Keywords:** cycling performance, ketoacidosis, ketone ester, muscle glycogen

## Abstract

Available evidence indicates that ketone bodies inhibit glycolysis in contracting muscles. Therefore, we investigated whether acute exogenous ketosis by oral ketone ester (KE) intake early in a simulated cycling race can induce transient glycogen sparing by glycolytic inhibition, thereby increasing glycogen availability in the final phase of the event. In a randomized crossover design, 12 highly trained male cyclists completed a simulated cycling race (RACE), which consisted of 3-h intermittent cycling (IMT_180′_), a 15-min time trial (TT_15′_), and a maximal sprint (SPRINT). During RACE, subjects received 60 g carbohydrates/h combined with three boluses (25, 20, and 20 g) (*R*)-3-hydroxybutyl (*R*)-3-hydroxybutyrate (KE) or a control drink (CON) at 60 and 20 min before and at 30 min during RACE. KE intake transiently increased blood d-β-hydroxybutyrate to ~3 mM (range: 2.6–5.2 mM) during the first half of RACE (*P* < 0.001 vs. CON). Blood pH concomitantly decreased from approximately 7.42 to 7.36 (range: 7.29–7.40), whereas bicarbonate dropped from 26.0 to 21.6 mM (range: 20.1–23.7; both *P* < 0.001 vs. CON)_._ Net muscle glycogen breakdown during IMT_180′_ [KE: −78 ± 30 (SD); CON: −60 ± 22 mmol/kg wet wt; *P* = 0.08] and TT_15′_ (KE: −9 ± 18; CON: −18 ± 18 mmol/kg wet wt; *P* = 0.35) was similar between KE and CON. Accordingly, mean power output during TT_15′_ (KE: 273 ± 38; CON: 272 ± 37 W; *P* = 0.83) and time-to-exhaustion in the SPRINT (KE: 59 ± 16; CON: 58 ± 17 s; *P* = 0.66) were similar between conditions. In conclusion, KE intake during a simulated cycling race does not cause glycogen sparing, nor does it affect all-out performance in the final stage of a simulated race.

**NEW & NOTEWORTHY** Exogenous ketosis produced by oral ketone ester ingestion during the early phase of prolonged endurance exercise and against the background of adequate carbohydrate intake neither causes muscle glycogen sparing nor improves performance in the final stage of the event. However, such exogenous ketosis may decrease buffering capacity in the approach of the final episode of the event. Furthermore, ketone ester intake during exercise may reduce appetite immediately after exercise.

## INTRODUCTION

Endurance exercise performance is largely determined by the ability of the body to generate sustained muscular work, which largely depends on muscular oxygen supply via cardiac output as well as ATP production via oxidative energy turnover (10, 13, 14). During continuous exercise, the energy to synthetize ATP is derived from the degradation of both intra- and extramuscular carbohydrate and lipid substrates, with only a trivial contribution of amino acids (41, 55). The balance between utilization of carbohydrates versus lipids is regulated by exercise intensity, with lower intensities resulting in higher fraction of fat utilization (41, 46). Furthermore, higher relative substrate availability also results in greater fractional utilization (30). From this perspective, numerous studies have explored the effect of changing substrate availability by dietary manipulation on endurance exercise performance (4, 27). These studies showed general improvements in performance with high-carbohydrate intake compared with consumption of large amounts of fat in events lasting ≥1 h (6, 48). Therefore, high-carbohydrate intake before and during exercise is still considered as the optimal choice to attain maximal endurance exercise performance (28).

Recent findings also have stimulated interest in the role of ketone bodies in exercise metabolism (12, 37). Ketone bodies (KB), i.e., d-β-hydroxybutyrate (βHB) and acetoacetate (AcAc), are produced by liver mitochondria in response to metabolic stressors that cause reduced carbohydrate availability such as starvation or prolonged exercise (23, 35). Under these conditions, KB provide an alternative oxidizable carbon source for energy provision in muscle, which, in turn, also increases the energy release from ATP hydrolysis compared with glucose or lipid oxidation (22, 24, 39, 43). Given the ability of KB to provide an alternative energy source, it may potentially cause sparing of finite glucose resources such as muscle glycogen during exercise. However, hyperketonemia by ketogenic diet fails to enhance endurance performance due to drop of carbohydrate availability (6, 25).

Nonetheless, exogenous ketosis produced by the ingestion of oral KB supplements, in the form of ketone salts, ketone precursors, or ketone esters, has emerged as a potential novel approach to impact endurance exercise performance via metabolic regulation. However, ingestion of ketone salts (34, 40, 59), ketone precursors (44, 45), or ketone diester (31) only marginally increased blood ketone concentrations ([βHB] < 1 mM) yet caused substantial gastrointestinal distress (31, 51). Conversely, oral intake of the ketone monoester (*R*)-3-hydroxybutyl (*R*)-3-hydroxybutyrate (KE) can significantly raise circulating βHB concentration during exercise (>3 mM), indeed, at low incidence of gastrointestinal complaints (11, 51). In a seminal study by Cox et al. (12), coingestion of ~40 g of KE and ~70 g of carbohydrates, compared with carbohydrate (~120 g) alone, slightly (~2%) improved 30-min time-trial performance following 1-h constant load cycling. This ergogenic effect was attributed to muscle glycogen sparing by inhibition of glycolytic flux in conjunction with stimulation of intramyocellular triglyceride oxidation. However, this trial was run in the fasted state, which shifts energy metabolism from carbohydrate to fat metabolism (15, 56) and, in fact, is not an adequate nutritional condition to study endurance exercise performance. Hence, a follow-up study with adequate carbohydrate intake before exercise observed no effect of KE on a 10-km run preceded by 1-h submaximal exercise (20). This study speculated that reduced glycolytic flux may rather reflect glycogenolytic impairment than sparing and may explain the lack of ergogenic effect in their exercise settings involving high-intensity efforts largely relying on carbohydrate utilization for energy provision (26).

Given the need for high-rate glycolytic ATP production in search of success in the final stage of most endurance cycling competitions, it is reasonable to postulate that KE supplementation should rather be used during the initial phase of an endurance event to spare muscle glycogen and thereby maintain the capacity for high glycolytic flux in the final episode of the race. However, to date, no studies have investigated whether such approach can enhance endurance exercise performance. Therefore, we investigated whether increasing blood ketone concentration by KE ingestion early in a cycling race inhibits glycogen degradation, indeed, and via this mechanism stimulates final high-intensity power outputs.

## METHODS

### 

#### Ethical approval and subjects.

Twelve highly trained, male cyclists and triathletes [age: 25 ± 6 (mean ± SD) yr; height: 1.77 ± 0.06 m; body mass: 72 ± 8 kg; maximal oxygen uptake rate (V̇o_2max_): 62.4 ± 6.6 mL·kg^−1^·min^−1^, range: 55–73 mL·kg^−1^·min^−1^; cycling activity: 11.4 ± 4.2 h/wk, range: 7–20 h/wk] participated in this study. Potential subjects were screened using a medical questionnaire and a physical examination, including a resting electrocardiogram, before involvement in the study. All participants were nonsmokers and did not take any medication or ergogenic supplement for ≥3 mo before the start of the study. They were instructed to maintain their habitual physical activity level and diet during the full study period but avoid strenuous exercise 48 h before each experimental session. The study was approved by the KU Leuven Biomedical Ethics Committee (B322201939080) and conforms to the Declaration of Helsinki. All subjects were informed of the content and potential risks involved with the experimental procedures before providing their written consent.

#### General study design.

The subjects performed two experimental sessions with a 1-wk washout period in between, in a double-blind, placebo-controlled, crossover design. Both experimental sessions were scheduled on the same time of the day and involved a simulated cycling race (RACE) consisting of 3-h submaximal intermittent cycling (IMT_180′_) followed by a 15-min time trial (TT_15′_) and an all-out SPRINT. Subjects received either 65 g (918 ± 102 mg/kg, range: 722–1,072 mg/kg) of KE [>96% (*R*)-3-hydroxybutyl (*R*)-3-hydroxybutyrate] or a viscosity- and taste-matched placebo (CON). Both were provided in three boluses (25, 20, and 20 g) to be ingested *1*) 60 and *2*) 20 min before IMT_180′_ and *3*) at *minute 30* of IMT_180′_, aiming to establish physiological ketosis (2–5 mM βHB) during the initial 1.5–2 h of IMT_180′_. KE was purchased from TdeltaS (Thame, Oxfordshire, United Kingdom), whereas the CON drink was prepared by dissolving collagen Peptan (12.5% wt/vol; 6d Sports Nutrition, Oudenaarde, Belgium) and 1 mM bitter sucrose octaacetate (Sigma-Aldrich, Bornem, Belgium) in water. Total energy content for the three doses administered was 306 kcal for KE versus 29 kcal for CON. Both were provided in nontransparent 50-mL tubes to avoid visual identification of the treatments. All exercise tests were conducted in an air-conditioned laboratory (18°C; 60% relative humidity) using the subjects’ own bicycle, which was mounted on a calibrated cycle ergometer (Avantronic Cyclus2, Leipzig, Germany).

#### Preliminary testing.

Two weeks before the first experimental session, the subjects participated in two familiarization trials with 4 days in between. On the first visit, they performed a maximal incremental-load cycling test to determine their lactate threshold (LT) as well as V̇o_2max_. Initial workload was set at 100 W, followed by 40-W increments per 8 min, until volitional exhaustion. Respiratory gas exchange was measured during the final phase of the test (Cortex MetaLyzer II; Leipzig, Germany) with V̇o_2max_ defined as the highest oxygen uptake measured over a 30-s period. Capillary blood samples were obtained from a hyperemic earlobe at *minutes 4* and *8* of each intensity block for lactate determination (Lactate Pro2; Arkray). LT was defined as the workload corresponding to a 1 mM blood lactate increment from *minute 4* to *8* within the same stage. Following a 15-min active recovery by cycling at 100 W, the subjects performed the final ~2 h of the simulated cycling race (see below) so as to reach an identical duration compared with the subsequent sessions. During the second visit, subjects completed the simulated cycling race as to be performed during the experimental trials.

#### Experimental sessions.

**Fig. 1.**
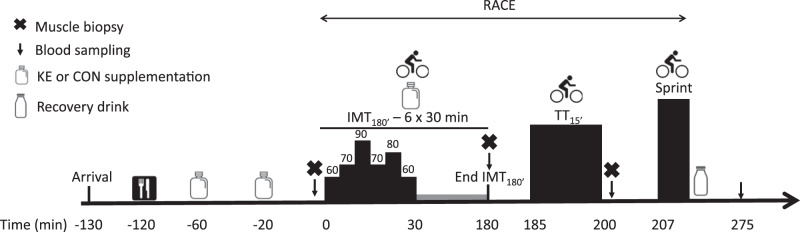
Schematic representation of the experimental sessions. In a double-blind, randomized, crossover design, subjects (*n* = 12) performed a simulated cycling race (RACE) consisting of 3-h intermittent cycling (IMT_180′_) followed by a 15-min time trial (TT_15′_) and a SPRINT. Before and during exercise, subjects received ketone ester (KE) or control (CON) in addition to optimal carbohydrate intake (60 g/h). Exercise intensity during IMT_180′_ was set relative to the lactate threshold of each individual.

The evening before both experimental sessions, the subjects reported to the laboratory for a standardized carbohydrate-rich dinner (~5,600 kJ; 69% carbohydrate, 16% fat, 15% protein). Next morning and following an overnight fast, they received a standardized breakfast (~2,600 kJ; 72% carbohydrate, 15% fat, 13% protein) followed by 500 mL of a 6% carbohydrate drink (6d Sports Nutrition) 90 min later. Two hours following breakfast, subjects started the RACE (Fig. 1). The exercise protocol started with IMT_180′_, which consisted of six identical 30-min blocks during which the intensity was varied per 5-min intervals between 60 and 90% of the lactate threshold (*minutes 0*–*5*: 60%; *minutes 5*–*10*: 70%; *minutes 10*–*15*: 90%; *minutes 15*–*20*: 70%; *minutes 20*–*25*: 80%; *minutes 25*–*30*: 60%). Throughout IMT_180′_, subjects ingested 500 mL of a 6% carbohydrate drink as well as an energy bar (6d Sports Nutrition) each hour, to obtain a carbohydrate intake of 60 g/h. Following IMT_180′_, subjects rested for 5 min before starting TT_15′_ in which they aimed for the highest possible mean power output. Workload during the initial 3 min (*t*_0_–*t*_3_) was set equal to the mean power output effected during the last familiarization session for TT_15′_. From *t*_3_ to *t*_12_, the subjects could adjust the workload at 3-min intervals according to their subjective perception of fatigue. During the last 3 min (*t*_12_–*t*_15_), 1-min adjustments were allowed to facilitate full exhaustion by the end of TT_15′_. On completion of TT_15′_, subjects rested for 5 min followed by 2-min cycling at 50 W, whereafter they started an all-out cycling bout at 175% of LT. Time to exhaustion was defined as drop of cadence to <70 revolutions/min. Five minutes after completion of the SPRINT, subjects received a 500-mL recovery drink delivering 60 g of carbohydrates and 30 g of proteins (6d Sports Nutrition), after which they rested for 1 h. Water was provided ad libitum during the first experimental session, and intake was recorded to allow replication during the second session. During IMT_180′_ and TT_15′_, but not during SPRINT, a countdown timer was shown but power output and heart rate were blinded to the subjects.

#### Assessment of perceived exertion, gastrointestinal distress, and appetite perception.

Ratings of perceived exertion (RPE) were evaluated halfway through IMT_180′_ and immediately after IMT_180′_, TT_15′_, and the SPRINT (6–20 Borg scale; Ref. 3). Gastrointestinal discomfort was assessed 6 min after completion of the SPRINT using a validated 0–8 Likert scale questionnaire, evaluating distress at the systemic (dizziness, headache, muscle cramp, urge to urinate), upper- (reflux, bloating, nausea, vomiting), and lower-abdominal (cramps, flatulence, abdominal pain, diarrhea) levels. Subsequently, appetite sensations were determined using a validated 10-point visual analog scale (60) assessing 4 appetite or satiety questions (“How hungry do you feel?”, “How full do you feel?”, “How satisfied do you feel?”, “How much do you think you could eat now?”).

#### Capillary blood sampling and analyses.

Blood d-β-hydroxybutyrate (βHB) and glucose concentrations were determined (GlucoMen LX plus meter with LX β-ketone and LX glucose strips; A. Menarini Diagnostics, Firenze, Italy) from capillary blood samples obtained immediately before and 30 min following ingestion of the first KE bolus as well as at 30-min intervals during IMT_180′_ and at the end of TT_15′_. To ensure double-blindness of the study, these measurements were performed by an investigator who otherwise was not involved in the experiments. Blood lactate concentration was assessed (Lactate Pro2; Arkray) in capillary blood obtained at regular intervals during IMT_180′_ and TT_15′_ and 5 min following the SPRINT. Furthermore, a 70-µl capillary blood sample from a hyperemic earlobe was obtained in a safeCLINITUBE (Radiometer Medical ApS, Copenhagen, Denmark) *1*) immediately before ingestion of the first KE bolus, *2*) at the start, *3*) middle, and *4*) end of IMT_180′_ *5*) as well as 3 min after completion of the SPRINT. Following hypoxic mixing for 10 s, acid-base balance, blood gasses, and plasma electrolytes were directly determined by an automated acid-base laboratory (ABL90 FLEX analyzer; Radiometer Medical ApS).

#### Venous blood sampling and analyses.

Venous blood samples were obtained from an antecubital vein (Venoject; Terumo, Tokyo, Japan) *1*) before, *2*) halfway through, and *3*) at the end of IMT_180′_, *4*) at the end of TT_15′_, and *5*) 1 h after exercise. Samples were collected into vacuum tubes containing either EDTA or Silica Clot Activator [Becton Dickinson (BD) Vacutainer]. Tubes were centrifuged (1,500 rpm for 10 min at 4°C), and the supernatant was stored at −80°C (EDTA plasma) or −20°C (serum) until later analysis. Commercially available ELISAs were performed to assess serum growth differentiation factor 15 (GDF15), leptin, and total ghrelin (GDF15: DGD150, R&D Systems; leptin: BMS2039INST, Thermo Fisher Scientific; total ghrelin: EZGRT-89K, Merck), whereas adrenaline and noradrenaline were determined in EDTA plasma [BA E-5400; Labor Diagnostika Nord (LDN), Nordhorn, Germany]. Plasma nonesterified free fatty acids (FFA) were determined using a colorimetric reagent kit (Wako Chemicals, Neuss, Germany). All samples per parameter were assayed in duplicate and in a single batch according to the respective manufacturer’s instructions. Coefficients of variation between the duplicates were 1.3 ± 1.2% for GDF15, 2.9 ± 2.8% for leptin, 1.7 ± 1.6% for ghrelin, 2.3 ± 3.0% for adrenaline, 2.6 ± 3.1% for noradrenaline, and 3.1 ± 3.9% for FFA.

#### Urine sampling and analyses.

Urine was collected starting from the first KE or CON bolus up to 1 h after exercise. Total urinary output volume was determined, and urinary ketone excretion was assessed using ketone reagent strips (Ketostix; Ascensia Diabetes Care).

#### Muscle biopsy procedure.

A percutaneous needle biopsy (100–200 mg) of the musculus vastus lateralis was taken under local anesthesia (2% xylocaine without adrenaline, 1 mL sc) immediately before exercise and after completion of IMT_180′_ and TT_15′_ using a 5-mm Bergström-type needle. To avoid interference between multiple biopsies (57), during the first experimental session, the preexercise biopsy was obtained from the left leg, whereas the biopsies at the end of IMT_180′_ and TT_15′_ were taken from the right leg with the needle pointing distal versus proximal, respectively. During the second experimental session, a similar procedure was followed using the other leg. Part of the muscle sample was immediately frozen in liquid nitrogen and stored at −80°C for biochemical assays at a later date. The remaining part was mounted in embedding medium (Tissue-Tek optimum cutting temperature), frozen in precooled isopentane, and kept at −80°C until histochemical analyses were done.

#### Muscle glycogen and intramyocellular triglyceride content.

Muscle glycogen content was determined in triplicate as glucose residues following acid hydrolysis using a standard enzymatic fluorometric assay (32). For determination of fiber type-specific intramyocellular triglyceride (IMTG) content, serial sections (7 µm) from m. vastus lateralis were laid together on uncoated glass slides. The staining procedure to quantify IMTG was performed using Oil red O (ORO), as previously described by our laboratory (15). Cryosections were fixed for 10 min using 0.1% Triton X-100 added to 4% paraformaldehyde in phosphate-buffered saline (PBS). After two 5-min washes (0.5% BSA in PBS), sections were treated with 10 mM NH_4_Cl to minimize autofluorescence and washed again (2 × 5 min). Following subsequent prehybridization with 1% BSA in PBS for 30 min, sections were incubated for 1 h at 37°C with primary antibodies directed against myosin heavy chain I (MHCI) and MHCIIa (BA-F8 and SC-71, respectively, both 1:200 in PBS; Developmental Studies Hybridoma Bank, Iowa City, IA). Following three 5-min washes, cryosections were incubated with the appropriate conjugated antibodies for 1 h at 37°C (type I: goat anti-mouse IgG2b Alexa Fluor 488, type IIa: goat anti-mouse IgG1 Cy5, both 1:500 in PBS; Jackson ImmunoResearch, West Grove, PA). Additionally, together with the secondary antibodies, membranes were stained using wheat germ agglutinin conjugated to Alexa Fluor 350 (Thermo Fisher Scientific). After three additional washes, sections were treated for 10 min with formalin and rinsed with deionized water (3 × 30 s). Thereafter, samples were treated with the ORO working solution for 15 min. The working solution was prepared by adding 20 mL of deionized water to 30 mL of the ORO stock solution [0.5 g of Oil red O (Sigma-Aldrich, St. Louis, MO) in 100 mL of 60% triethyl phosphate] and was filtered three times (prefilter: Millipore AP25; filter: Millex-HV 0.45 µm, Millipore) before application. On completion of the ORO procedure, sections were rinsed with deionized water (3 × 30 s) before being immersed under tap water for 7 min. Slides were mounted using Fluorescent Mounting Medium (DakoCytomation, Glostrup, Denmark). Samples were visualized by fluorescence microscopy (Nikon E1000; Nikon, Boerhavedorp, Germany) at ×40 magnification. Images were analyzed in a blinded manner resulting in a total of 115 ± 14 quantified muscle fibers per biopsy. Results are expressed as arbitrary units (AU).

#### Statistical analyses.

Statistical analyses were performed using GraphPad Prism version 8.3.0 (GraphPad Software, La Jolla, CA). A two-way repeated-measures analysis of variance (condition by time) was used to evaluate differences over time and between conditions. Whenever a significant interaction effect was detected, a post hoc analysis using Bonferroni correction was incorporated into the repeated-measures analysis of variance. In case of a significant interaction effect, *P* values refer to the output of the post hoc analyses, but otherwise *P* values for main effects were reported. Alterations between conditions at one time point were assessed using a paired *t* test. Correlations were calculated using a two-tailed Pearson test. Statistical significance was set at *P* < 0.05. All data are expressed as means ± SD. Ninety-five percent confidence intervals (CI) were included in the text for the primary outcome variables.

## RESULTS

### 

#### Blood βHB concentration and urinary ketone excretion.

**Fig. 2.**
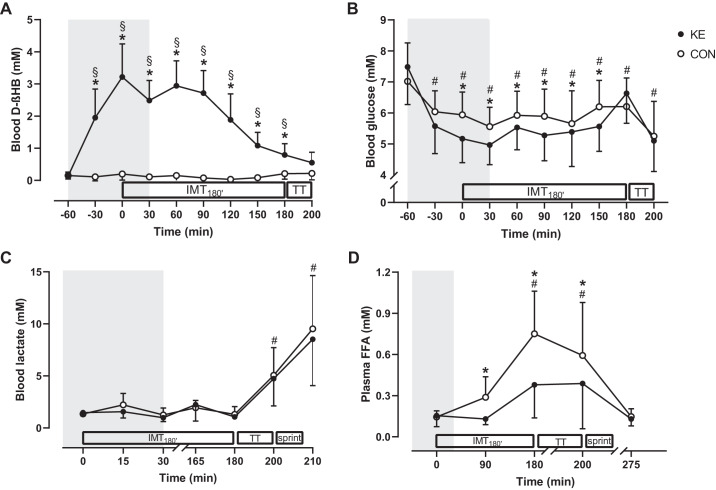
Effect of ketone ester supplementation on blood d-βHB, glucose, lactate, and plasma free fatty acid concentrations. Data are means ± SD for blood d-β-hydroxybutyrate (d-βHB; *A*), glucose (*B*), lactate (*C*), and plasma free fatty acid (FFA; *D*) concentrations during each trial. Before and at the beginning of the exercise protocol, subjects received control (CON; ○; *n* = 12) or ketone ester supplements (KE; ●; *n* = 12). Two-way repeated-measures ANOVA with a Bonferroni post hoc test was used. *P < 0.05, KE vs. CON at time points indicated; #*P* < 0.05 vs. baseline for both KE and CON; §*P* < 0.05 vs. baseline for the indicated condition. IMT_180′_, 3-h submaximal intermittent cycling; TT, 15-min time trial. Gray area indicates time zone during which subjects received KE or CON.

Blood βHB concentrations at baseline were low (approximately 0.1–0.4 mM) in both KE and CON (*P* = 0.92; Fig. 2*A*) and remained low in CON throughout the trial. Following KE ingestion, blood βHB increased to peak level within 30 min, causing a stable physiological ketosis of approximately 2–3 mM during the initial 2 h of IMT_180′_ (*P* < 0.001 vs. baseline). Thereafter, blood βHB gradually dropped to <1 mM by the start of TT_15′_ and SPRINT (TT_15′_: 0.8 ± 0.4 mM; SPRINT: 0.6 ± 0.3 mM). Ketone bodies were undetectable (<0.05 g/L) in urine in CON, whereas in KE a small fraction was excreted (0.2 ± 0.1 g; *P* < 0.001). Urine volume on average was lower in KE (300 ± 264 mL) than in CON (522 ± 402 mL; *P* = 0.04).

#### Blood glucose, lactate, and plasma free fatty acid concentrations.

Baseline blood glucose concentration was similar between KE (7.5 ± 1.2 mM) and CON (7.0 ± 1.2; *P* = 0.23; Fig. 2*B*). KE ingestion decreased blood glucose, resulting in ~0.5 mM lower concentration throughout the initial 150 min of IMT_180′_ in KE versus CON (*P* < 0.05). Thereafter, blood glucose concentrations were similar between conditions. Blood lactate concentrations during IMT_180′_ and TT_15′_ as well peak lactate concentrations following the SPRINT (KE: 8.5 ± 4.5 vs. CON: 9.5 ± 5.1 mM; *P* = 0.37; Fig. 2*C*) were similar between KE and CON. Baseline plasma FFA was similar between the experimental conditions at ~0.15 mM (*P* = 0.99; Fig. 2*D*). Plasma FFA gradually increased during RACE in both conditions (*P* < 0.001), yet the rise was 50% lower in KE compared with CON. Thus plasma FFA concentrations were lower in KE compared with CON by the end of IMT_180′_ (KE: 0.38 ± 0.24 vs. CON: 0.75 ± 0.31 mM, 95% CI of difference: −0.52 to −0.22 mM; *P* < 0.001) and TT_15′_ (KE: 0.39 ± 0.33 vs. CON: 0.59 ± 0.39 mM, 95% CI of difference: −0.36 to −0.05 mM; *P* < 0.01). One hour after exercise, plasma FFA concentrations had returned to baseline values in both conditions.

#### Exercise performance and RPE.

**Fig. 3.**
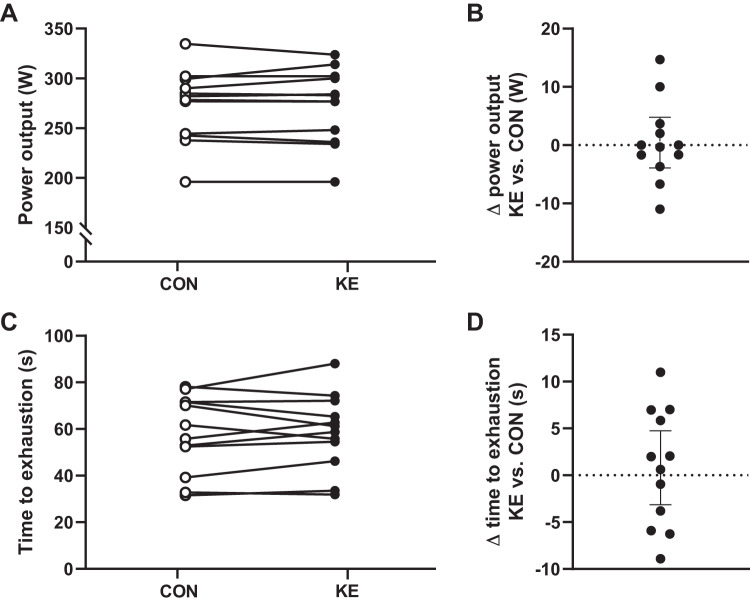
Effect of ketone ester supplementation (KE) on exercise performance. Individual data points for mean power output during the 15-min simulated time trial (TT_15′_; *A*) and difference in mean power output during TT_15′_ for each individual between the KE and control (CON) condition (*B*) are shown. Individual data points for time to exhaustion in the SPRINT test (*C*) and difference in time to exhaustion in the SPRINT test for each individual between the KE and CON condition (*D*) are shown. Subjects received either control (○; *n* = 12) or ketone ester supplements (●; *n* = 12). *B* and *D*: points above dotted line favor KE. Paired *t* test was used.

Mean power output during TT_15′_ (KE: 273 ± 38 vs. CON: 272 ± 37 W, 95% CI of difference: −5 to +4 W; *P* = 0.83; Fig. 3*A*) and time to exhaustion in the SPRINT (KE: 59 ± 16 vs. 58 ± 17 s, 95% CI of difference: −5 to +4 s; *P* = 0.66; Fig. 3*B*) were identical between conditions. Performance outcomes were also similar between the first and second experimental session for TT_15′_ (95% CI of difference: −5 to +4 W; *P* = 0.74) and SPRINT (95% CI of difference: −4 to +4 s; *P* = 0.88), thus excluding a potential order effect. Rate of perceived exertion tended to be higher in KE than in CON halfway through IMT_180′_ (KE: 13.3 ± 2.0 vs. CON: 12.3 ± 1.1, 95% CI of difference: −0.01 to + 2.02; *P* = 0.06), but not in the final stage of the trial.

#### Muscle glycogen and intramyocellular triglyceride content.

**Fig. 4.**
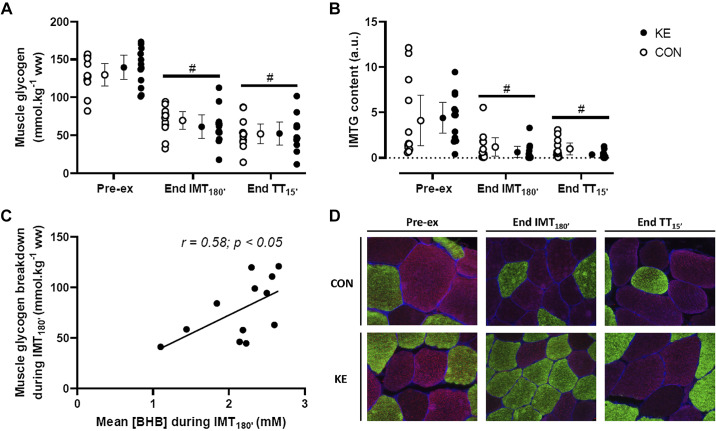
Effect of ketone ester on muscle glycogen and intramyocellular triglyceride content and relationship between blood d-β-hydroxybutyrate (BHB) and glycogen breakdown. Data are means ± 95% confidence intervals together with individual data points for muscle glycogen (*A*) and intramyocellular triglyceride (IMTG) content (*B*) before (Pre-ex) and immediately after 3-h submaximal intermittent cycling (IMT_180′_) and a 15-min time trial (TT_15′_). *C*: correlation analyses showing a positive correlation between mean blood BHB concentrations and muscle glycogen breakdown during IMT_180′_. *D*: representative immunofluorescence images of the Oil red O staining for determination of IMTG content of musculus vastus lateralis. Intramyocellular lipid droplets (red) and type I (green), type IIa (blue), and type IIx (black) fibers are shown. Subjects received either control (CON; ○; *n* = 12) or ketone ester supplements (KE; ●; *n* = 12). Two-way repeated-measures ANOVA with a Bonferroni post hoc test was used. #*P* < 0.05 vs. baseline for both KE and CON. a.u., Arbitrary units; ww, wet weight.

Initial muscle glycogen content was 139 ± 25 mmol/kg wet wt in KE versus 130 ± 23 mmol/kg in CON (*P* = 0.47; Fig. 4*A*). IMT_180′_ tended to decrease muscle glycogen concentration more in KE (minus 78 ± 30 mmol/kg wet wt) than in CON (minus 60 ± 22 mmol/kg wet wt, 95% CI of difference: −38 to +2 mmol/kg wet wt; *P* = 0.08). Nonetheless, muscle glycogen store at the start of TT_15′_ was similar between conditions (KE: 61 ± 24 vs. CON: 69 ± 19 mmol/kg wet wt, 95% CI of difference: −26 to + 9 mmol/kg wet wt; *P* = 0.70). TT_15′_ further decreased muscle glycogen content (KE: minus 9 ± 18 vs. CON: minus 18 ± 18 mmol/kg wet wt; *P* = 0.35), resulting in identical glycogen contents at the start of the SPRINT (KE: 52 ± 24 vs. CON: 52 ± 21 mmol/kg wet wt, 95% CI of difference: −17 to +18 mmol/kg wet wt; *P* = 0.99). IMTG content was similar between the conditions at baseline (KE: 4.4 ± 2.7 vs. CON: 4.1 ± 4.4 AU, 95% CI of difference: −2 to +3; *P* = 0.99; Fig. 4*B*). IMTG breakdown during IMT_180′_ (–3.8 ± 2.1 vs. – 2.9 ± 3.4, 95% CI of difference: −2.9 to +1.2; *P* = 0.61) and SPRINT (–0.3 ± 0.9 vs. – 0.2 ± 1.1, 95% CI of difference: −2.1 to +2.0; *P* = 0.99) were similar between KE vs. CON, respectively. Mean blood βHB concentrations during IMT_180′_ in KE were positively correlated with the degree of muscle glycogen breakdown (*r* = 0.58, *P* < 0.05; Fig. 4*C*), indicating higher rate of exercise-induced net glycogenolysis at higher circulating βHB. Conversely, plasma FFA concentrations at the end of IMT_180′_ were negatively correlated with the extent of muscle glycogen breakdown during IMT_180′_ (*r* = −0.45, *P* = 0.03).

#### Acid-base balance and blood gases.

**Fig. 5.**
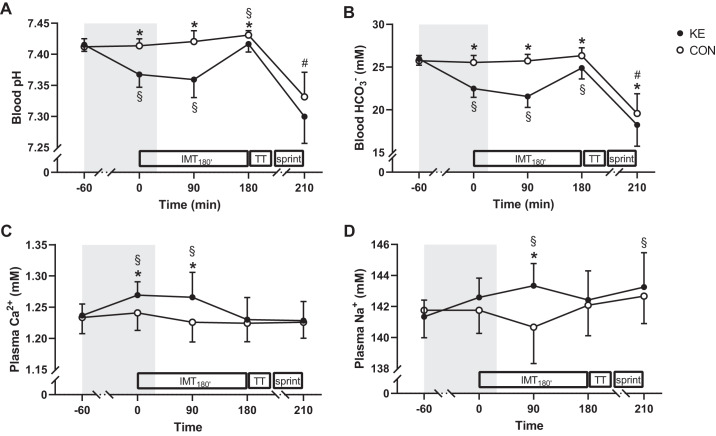
Effect of ketone ester supplementation on blood acid-base balance and plasma electrolytes. Data are means ± SD for blood pH (*A*) and bicarbonate (${\text{HCO}}_{3}^{-}$; *B*) and plasma calcium (Ca^2+^; *C*) and sodium (Na^+^; *D*) concentration at baseline (−60 min) and immediately before (0 min), during (90 min), and after (180 min) 3-h submaximal intermittent cycling (IMT_180′_) and immediately after the SPRINT (210 min). During the experimental trials, subjects received either control (CON; ○; *n* = 12) or ketone ester supplements (KE; ●; *n* = 12). Two-way repeated-measures ANOVA with a Bonferroni post hoc test was used. *P < 0.05, KE vs. CON at time points indicated; #*P* < 0.05 vs. baseline (−60 min) for both KE and CON; §*P* < 0.05 vs. baseline (−60 min) for the indicated condition. Gray area indicates time zone during which subjects received KE or CON.

Blood pH and [${\text{HCO}}_{3}^{-}$] at baseline were similar between conditions (pH: 7.42 ± 0.01 vs. 7.41 ± 0.01; [${\text{HCO}}_{3}^{-}$]: 26.0 ± 0.7 vs. 25.7 ± 0.6 mM for KE vs. CON; both *P* > 0.05; Fig. 5, *A* and *B*). Following KE intake, blood pH (7.37 ± 0.02) and [${\text{HCO}}_{3}^{-}$] (22.5 ± 1.0 mM) slightly dropped, resulting in lower values compared with CON throughout IMT_180′_ (*P* < 0.05). At the end of the SPRINT, blood pH and [${\text{HCO}}_{3}^{-}$] were decreased to a similar extent in both conditions (*P* > 0.05). The KE-induced pH drop was associated with a lower Pco_2_ halfway through (−7 ± 8%) IMT_180′_ for KE versus CON (*P* < 0.001). There were no differences between the experimental conditions for the partial pressure of O_2_ (Po_2_).

#### Plasma electrolytes.

Plasma calcium, sodium, chloride, and potassium concentrations were similar between KE and CON at baseline. Plasma calcium concentrations were ~0.3 mM higher in KE compared with CON at the start and halfway through IMT_180′_ (both *P* < 0.001; Fig. 5*C*). Plasma sodium concentrations were 2.7 ± 2.5 mM higher in KE compared with CON halfway through IMT_180′_ (*P* < 0.001; Fig. 5*D*). Neither plasma chloride nor potassium concentrations were different between KE and CON at any time point.

#### Hormonal parameters.

**Fig. 6.**
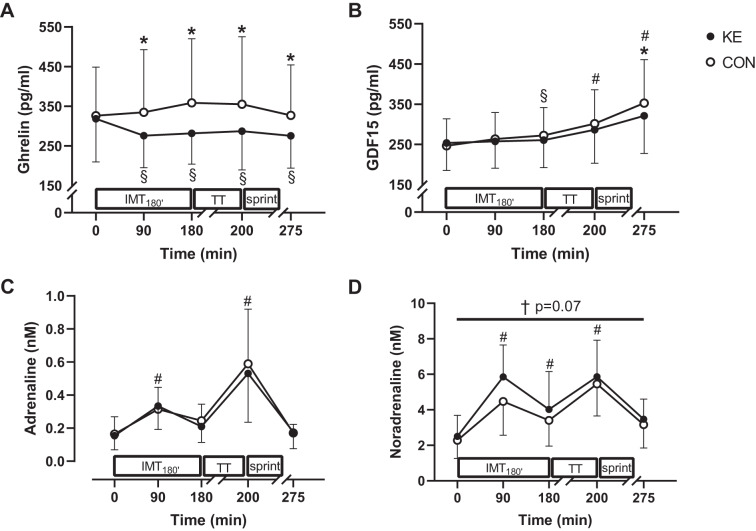
Effect of ketone ester supplementation on blood hormones. Data are means ± SD for serum ghrelin (*A*) and growth differentiation factor 15 (GDF15; *B*) and plasma adrenaline (*C*) and noradrenaline (*D*) before (0 min), during (90 min), and after (180 min) 3-h submaximal intermittent cycling (IMT_180′_), immediately after the 15-min time trial (TT; 200 min), and following a 1-h recovery period (275 min). Subjects received control (CON; ○; *n* = 12) or ketone ester supplements (KE; ●; *n* = 12). Two-way repeated-measures ANOVA with a Bonferroni post hoc test was used. *P < 0.05, KE vs. CON at time points indicated; #*P* < 0.05 vs. before exercise (pre-ex; 0 min) for both KE and CON; §*P* < 0.05 vs. pre-ex (0 min) for the indicated condition; †*P* = 0.07, KE vs. CON at time points indicated.

We assessed serum hormones implicated in regulation of energy homeostasis and appetite (i.e., GDF15, ghrelin, and leptin) as well as catecholamines (i.e., adrenaline and noradrenaline). All hormonal parameters measured were similar between experimental conditions at baseline (Fig. 6). Compared with baseline, RACE gradually increased GDF15 until 1 h after exercise (*P* < 0.001 vs. baseline). However, compared with CON, the exercise-induced increment of serum GDF15 was 10% smaller in KE. Thus at 1 h after RACE GDF15 was ~9% lower in KE (321 ± 94 pg/mL) than in CON (353 ± 108 pg/mL; *P* < 0.01). Serum ghrelin was unaffected by the exercise in CON. However, in KE, ghrelin rapidly dropped, yielding circulating concentrations at ~275 pg/mL throughout the trial and ~17% lower than CON (*P* < 0.05 at all time points). Serum leptin and plasma adrenaline concentrations were affected neither by the exercise protocol nor by KE. Compared with CON, however, noradrenaline concentrations tended to be slightly higher in KE (main effect of condition: *P* = 0.07).

#### Appetite sensations and gastrointestinal tolerance.

KE ingestion decreased the perception of hunger (KE: 3.6 ± 1.2 vs. CON: 4.9 ± 2.2; *P* = 0.03) as well as the desire to eat (KE: 5.1 ± 1.6 vs. CON: 6.5 ± 1.8; *P* < 0.01) after exercise, whereas no differences were observed for the perceived fullness and nutritional satisfaction. Total gastrointestinal discomfort scores were small in both experimental conditions (KE: 13 ± 10 vs. CON: 12 ± 12 out of 96; *P* = 0.52). Lower-abdominal (~3 out of 32) and systemic (~6 out of 32) distress were similar between the conditions, whereas the incidence of upper-abdominal discomfort was slightly higher in KE (KE: 4 ± 3 vs. CON: 2 ± 3 out of 32; *P* = 0.03).

#### Identification of condition and best performance trial.

On completion of the second experimental session, the subjects were asked to identify the order of their experimental conditions (KE vs. CON) and their best TT_15′_ performance. Seven of the twelve participants (58%) correctly identified their experimental conditions. However, all subjects were doubtful about their choice (certainty of response: 4 ± 2 out of 10; 0: no idea at all; 10: absolutely certain). Six subjects (50%) correctly identified their trial with the best performance on the TT_15′_. These data indicate that the blinding of the conditions was successful.

## DISCUSSION

Ketosis-induced glycogen sparing conceivably may be ergogenic in prolonged endurance exercise events wherein a low-to-moderate-intensity initial phase is followed by a high-intensity final part involving intermittent short maximal exercise bouts and sprinting. Therefore, in the present study, we investigated whether repeated oral ketone ester ingestion (KE) during the first half of a simulated cycling race (RACE) could *1*) inhibit net muscle glycogen breakdown and *2*) facilitate high power production in the final part of the event. It is well established indeed that residual muscle glycogen availability at the end of an endurance exercise event is an important determinant of the capacity to perform additional short maximal exercise (33). However, contrary to our hypothesis, in the conditions of the current study, KE neither reduced muscle glycogen breakdown nor improved performance in a 15-min time trial or a constant-load sprint at the end of the event. In fact, we rather demonstrate that KE, by causing transient metabolic acidosis, may result in reduced extracellular buffering capacity in the approach of the final stage of the race.

It has been suggested that acute exogenous ketosis during exercise causes upregulation of IMTG oxidation versus downregulation of muscle glycogenolysis in energy metabolism (16, 19). However, this opinion is based on observations in a single study where in a 2-h constant-load exercise bout at 70% V̇o_2max_ was performed after an overnight fast, i.e., in the absence of a usual carbohydrate-rich preexercise meal. Furthermore, the rate of carbohydrate intake during exercise was ~40% higher in the control experiments (1.2 g/min) than during ketosis (0.7 g/min). However, the rate of carbohydrate intake during exercise intake per se is a pivotal factor in regulating the fractional contribution of carbohydrates, including muscle glycogen, versus fats in energy metabolism (26, 29, 54). In addition, muscle glycogen concentration was measured by a semiquantitative method (periodic acid-Schiff staining), which lacks specificity for glycogen (47) and only yields relative muscle glycogen values. In addition, interassay variation and sample-freezing artifacts may impair the validity of repeated-measures comparisons. Therefore, in the current study, we measured mixed muscle glycogen concentrations with a quantitative enzymatic assay. In addition, we reproduced the recommended nutritional background for optimal endurance exercise performance (5), i.e., a carbohydrate-rich meal delivering approximately 1–4 g CHO/kg body wt approximately 1–4 h before the event, as well as an identical high-rate carbohydrate intake during exercise (60 g/h) in both KE and CON.

In this nutritional context, KE impacted neither the magnitude of net muscle glycogen breakdown nor IMTG utilization during IMT_180′_ or TT_15′_. However, KE markedly inhibited the rise in plasma FFA during RACE, which probably resulted from βHB-induced inhibition of adipolysis via activation of the nicotinic acid receptor PUMA-G (53). In addition, KE did not improve performance in TT_15′_ or SPRINT. Surprisingly, in KE, higher circulating blood βHB was associated with more net glycogen breakdown during IMT_180′_ (*r* = 0.58). This was likely caused by βHB-induced suppression of plasma FFA content, resulting in less inhibition of muscle glycogenolysis by FFA (18, 42). In support of this hypothesis, lower plasma FFA concentrations correlated with increased glycogen breakdown during IMT_180′_ (*r* = −0.45). Collectively, our findings thus clearly indicate that in the current experimental conditions involving adequate carbohydrate supply to stimulate cycling performance, KE affected neither intramuscular energy substrate use nor performance. This suggests that the glycogen sparing effect and concomitant performance benefit due to KE seen by Cox et al. (12) conceivably were due to the specific nutritional conditions used in their protocol because these effects were entirely negated by following recommended rates of carbohydrate intake before and during exercise in both the absence and presence of KE intake in the current study.

Besides the purported effect of KE on glycolytic rate (12), there is clear evidence to indicate that KE might also impact exercise performance by altering blood acid-base balance. Ketone ester ingestion at the dosages commonly recommended (20- to 30-g boluses) causes transient metabolic acidosis (17, 50) by increasing circulating βHB and AcAc concentrations for approximately 2–3 h after ingestion (9, 50). Such effect is likely to impair high-intensity exercise performance (7). Accordingly, the repeated ingestion of a ketone ester supplement before and early in RACE (65-g KE over a 90-min period) gradually decreased blood pH from 7.42 to 7.37. Blood ${\text{HCO}}_{3}^{-}$ content concomitantly dropped by ~15%. However, after cessation of the ketone ester intake, blood pH and ${\text{HCO}}_{3}^{-}$ content returned to normal during the second half of RACE. Still, compared with CON by the start of TT_15′_, pH and [${\text{HCO}}_{3}^{-}$] were slightly lower in KE. However, this did not significantly affect either blood lactate or pH and [${\text{HCO}}_{3}^{-}$] drop or performance in either TT_15′_ or SPRINT. This is in agreement with a previous study showing that compensation mechanisms (e.g., hyperventilation and decrease in Pco_2_) are sufficient to counteract potential ergolytic effects induced by the acid load of KE (17). Nonetheless, KE tended to cause higher RPE values during the initial phase of RACE, which is in apparent contrast with previous studies showing no effect of KE on RPE (20, 21). This discrepancy probably results from differences in the exercise context. Faull et al. (21) assessed RPE during a maximal incremental-load cycling test, suggesting that KE does not affect RPE during short high-intensity exercise. Furthermore, Evans et al. (20) found exogenous ketosis not to alter RPE during a 1-h constant-load running bout (65% of V̇o_2max_) followed by a 10-km all-out running test. However, in the conditions of this study, blood βHB levels during exercise were low (<1.5 mM). The above literature data taken together with the current observations may suggest that KE increases RPE, indeed, during prolonged low-intensity exercise associated with high (>2 mM) blood βHB concentrations. Support for such interpretation comes from an earlier study showing that leg discomfort during cycling was increased by KE at submaximal workloads, but not during maximal exercise, and that the extent of this effect was positively associated with the degree of ketoacidosis (21).

A recent study indicated that KE, compared with isocaloric glucose ingestion, acutely suppresses hunger and the desire to eat in healthy subjects at rest (49). Given the importance of adequate food intake immediately following exercise to stimulate muscle repair and recovery (1), we also evaluated the effect of KE on appetite regulation in the conditions of the current study. In line with earlier observations in healthy subjects at rest (49), here we add the observation that KE intake during exercise suppressed both hunger perception and the desire to eat immediately after exercise. Attempting to explain this KE-induced appetite shift, we also measured the so-called “appetite hormones.” By analogy with the earlier findings by Stubbs et al. (49), KE rapidly and consistently suppressed serum ghrelin concentrations, whereas serum leptin was unchanged. This probably indicates that acute appetite regulation during intermittent exogenous ketosis effected by ketone ester intake is at least partly effected by ghrelin and is independent of leptin. Interestingly, we (38) recently also demonstrated that KE during a period of training overload eventually increased spontaneous food intake to compensate for elevated energy expenditure in training, probably by short-term downregulating of GDF15 as neither ghrelin nor leptin was affected. Available literature indeed indicates that GDF15 suppresses food intake during episodes of metabolic stress (e.g., high-fat feeding; Ref. 36). Accordingly, here we report the novel finding that KE blunted the acute exercise-induced rise in GDF15. Such effect eventually might also explain the lower baseline GDF15 levels seen during rapid succession of training sessions with short rest intervals in between, eventually explaining higher rate of spontaneous energy intake during training overload as we (38) have recently reported. Taken together, our current and earlier data (38) provide evidence to indicate that GDF15 is an important long-term modulator of appetite during training. Conversely, ghrelin is probably more important in acute appetite regulation by KE, both in the absence (49) and presence of acute exercise.

Concerns have been raised about the gastrointestinal side effects induced by oral ketone supplementation in conjunction with exercise (31, 58). However, previous studies have indicated that gastrointestinal complaints are less frequent with the ketone monoester (*R*)-3-hydroxybutyl (*R*)-3-hydroxybutyrate than with available ketone salts or the diester 1,3-butanediol acetoacetate (51, 52) and are similar to the discomfort due to ingestion of carbohydrate drinks during exercise (51). In the conditions of the current study, gastrointestinal discomfort scores were very low and were identical between KE and CON, indicating that KE usage in conjunction with high-rate carbohydrate intake during exercise in general is well tolerated.

Another novel observation in the current study is that KE ingestion markedly suppressed urine production. Against the background of standardized fluid intake before and during exercise (616 ± 78 mL/h, range: 508–732 mL/h), compared with CON, urinary output from the start of KE intake until 1 h after exercise was reduced by ~40%. The precise physiological mechanism underlying this antidiuretic effect remains to be elucidated. Interestingly, however, we observed KE to slightly raise plasma sodium concentrations halfway through IMT_180′_. Such effect most probably is due to facilitated renal tubular sodium absorption. Support for such assumption comes from a study in diabetic rats showing elevated βHB during ketoacidosis to stimulate vasopressin secretion (8). Vasopressin and the renin-angiotensin-aldosterone system are the primary regulators of renal tubular sodium and water coabsorption (2). Future studies must address the impact of exogenous ketosis on regulation of the antidiuretic hormonal axis during exercise.

In conclusion, this study clearly demonstrates that exogenous ketosis during a simulated cycling race affects neither intramyocellular triglyceride or muscle glycogen breakdown nor high-intensity exercise performance in the final stage of the event. However, the low degree of transient ketoacidosis induced by ketone ester intake may increase fatigue perception during low-intensity episodes of a race. Finally, high-rate ketone ester intake during exercise may blunt postexercise appetite via ghrelin suppression. Therefore, the current findings and available literature data taken together do not support an ergogenic action of oral KE intake in endurance exercise whenever good practice with regard to carbohydrate intake before and during exercise is in place. To allow further identification of the potential benefits or drawbacks of ketone supplements for athletes, future studies need to incorporate research designs that are relevant for real-life exercise events as well as should include adequate nutritional conditions being recommended for optimal performance.

## GRANTS

This study was funded by Research Fund Flanders (Fonds voor Wetenschappelijk Onderzoek – Vlaanderen; Research Grant G080117N).

## DISCLOSURES

No conflicts of interest, financial or otherwise, are declared by the authors.

## AUTHOR CONTRIBUTIONS

C.P. and P.H. conceived and designed research; C.P., M.R., and S.B. performed experiments; C.P. and P.H. analyzed data; C.P. and P.H. interpreted results of experiments; C.P. prepared figures; C.P. and P.H. drafted manuscript; C.P., S.B., and P.H. edited and revised manuscript; C.P., M.R., S.B., and P.H. approved final version of manuscript.
